# Three new species of *Rugitermes* (Isoptera, Kalotermitidae) from Peru and Bolivia

**DOI:** 10.3897/zookeys.1000.59219

**Published:** 2020-12-03

**Authors:** Rudolf H. Scheffrahn, Tiago F. Carrijo

**Affiliations:** 1 University of Florida, Fort Lauderdale Research & Education Center, 3205 College Avenue, Davie, Florida 33314, USA University of Florida Davie United States of America; 2 Centro de Ciências Naturais e Humanas, Universidade Federal do ABC, Rua Arcturus 03, Jardim Antares, 09606‐070 São Bernardo do Campo, SP, Brazil Universidade Federal do ABC São Bernardo do Campo Brazil

**Keywords:** Amboró National Park, barcode, coloration, GMYC analysis, Huánuco, imago, soldier

## Abstract

The soldier of *Rugitermes aridus***sp. nov.** is described from a xeric, termite-depauperate region of central Peru. *Rugitermes rufus***sp. nov.** and *R. volcanensis***sp. nov.** are described from soldiers and dealated imagos collected in a mesic forest of Amboró National Park in western Bolivia. The imago of *R. rufus* is unique among all described *Rugitermes* species in that the head capsule is reddish orange and the pronotum is brown. The imago head and pronotum are both brown in *R. volcanensis*. A phylogenetic and GMYC barcode analyses were performed with the COI gene. These analyses confirmed the three new species and revealed a high undescribed diversity of *Rugitermes* in the New World.

## Introduction

*Rugitermes* Holmgren, 1911 now consists of 14 extant species (Krishna et al. 2013; Scheffrahn 2015; Scheffrahn and Florian 2020). *Rugitermes* has a non-insular Neotropical distribution with exception of the French Polynesian *R. athertoni* (Light, 1932) and species on the continental islands of Trinidad and Tobago (Scheffrahn 2019a, 2019b). The genus *Rugitermes* differs from other kalotermitid genera by the imago’s basal confluence of the median vein into the radial vein and the soldier’s anterolateral ridges lateral to the frons. In nine *Rugitermes* species, the imago has a striking color contrast between the blackish head and the yellowish pronotum, a combination not known in any other Neotropical kalotermitid genus. Until now, only *R. laticollis* Snyder, 1957 has been reported from Bolivia (Scheffrahn 2015), and only *R. magninotus* (Emerson, 1925) is known from Peru (Scheffrahn 2019b).

**Figure 1.**
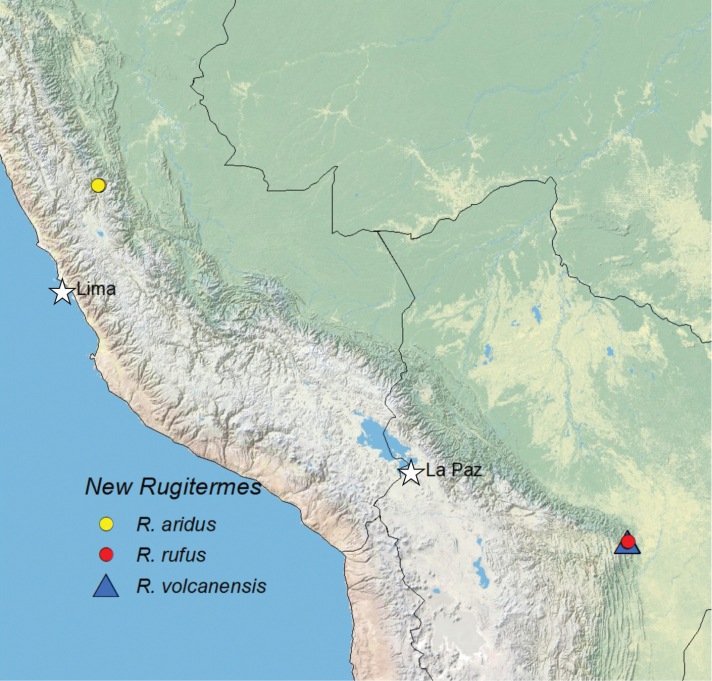
Localities of the new *Rugitermes* species described herein.

Herein we describe the soldier caste of *R. aridus* from Peru and the dealated imago and soldier castes of *Rugitermes rufus* and *R. volcanensis* from Bolivia (Fig. 1).

## Material and methods

Photographs of soldiers and imagos were taken as multi-layer montages using a Leica M205C stereomicroscope controlled by Leica Application Suite v. 3 software. Preserved specimens were taken from 85% ethanol and suspended in a pool of Purell Hand Sanitizer to position the specimens on a transparent plastic Petri dish as background. All soldiers and imago specimens listed in “material examined” sections were used for measurements (Table 1 and Table 2, respectively).

DNA extraction, PCR, and the sequencing of 43 *Rugitermes* specimens were performed at the Canadian Centre for DNA Barcoding (BOLD) following standard high-throughput protocols (deWaard et al. 2008). The PCR employed the primers LepF1 and LepR1 (Hebert et al. 2003) which generated 558–658 bp of the barcode region of the mitochondrial gene cytochrome c oxidase subunit 1 (COI). As outgroups for the phylogenetic analysis, we used a sequence of *Incisitermes bequaerti* (Snyder, 1929) from BOLD (BIN:ADD6661) and one of *Zootermopsis angusticollis* (Hagen, 1858) from GenBank (accession no. JX144932).

All sequences were aligned using the MUSCLE algorithm in Geneious v. 9.1.8 (Biomatters Ltd, Auckland, New Zealand). A phylogenetic analysis was conducted under Bayesian inference (BI) with BEAST v. 1.8.0 (Drummond et al. 2012). The substitution model used (GTR+I+G) was selected through the Akaike Information Criterion (AIC) with the jModelTest2 (Darriba et al. 2012). A Yule speciation process, with a random starting tree, and strict clock were used as tree priors. Tree independent Markov chain Monte Carlo (MCMC) searches were conducted for 70,000,000 generations and combined. Convergence and stationarity were assessed with Tracer v. 1.5 (Rambaut et al. 2014) and the first 10% of trees were discarded as burn-in.

To infer the number of *Rugitermes* species in our COI tree, a maximum likelihood version of the Generalized Mixed Yule Coalescent model (GMYC) was used. The GMYC tests whether the diversification history is better explained under a population coalescent model or under a speciation model (e.g., Yule 1925). It does that by detecting clustering beyond levels expected in a null model that all sampled individuals belong to a single population (Fujisawa and Barraclough 2013). This analysis was conducted with the R package splits with the single threshold method (Fujisawa and Barraclough 2013), in R v. 3.4.3 (R Core Team 2018).

### Key to *Rugitermes* from Bolivia and Peru

**Table:** 

1	Maximum soldier head width (mean) ca 2 mm; imago head capsule, pronotum, and distal antennal articles black	***R. laticollis* Snyder**
–	Maximum soldier head width (mean) <1.7 mm	**2**
2	Imago head black, pronotum yellowish orange (Fig. 7, new record for Bolivia	***R. magninotus* Emerson**
–	Imago unknown or imago pronotum brown	**3**
3	Imago unknown; soldier head capsule width at antenna narrower than span of antennal carinae; soldier antennal carinae and anterolateral ridges smoothly rounded and appear polished (Fig. 2B); xeric habitat	***R. aridus* sp. nov.**
–	Soldier head width at antennal carinae equal to span of antennal carinae; soldier antennal carinae and anterolateral ridges angular and rugose (Figs 5B, 8B); mesic forest habitat	**4**
4	Imago head capsule reddish orange (Figs 4A, B, 6)	***R. rufus* sp. nov.**
–	Imago head capsule brown (Figs 4C, D, 7)	***R. volcanensis* sp. nov.**

## Taxonomy

### Rugitermes aridus

Taxon classificationAnimaliaIsopteraKalotermitidae

Scheffrahn sp. nov.

5A6DDDB0-1611-5031-A9FD-390E9DC1A460

http://zoobank.org/B347F7B3-ADE0-4211-B26A-D0AF5FED9427

**Figure 2.**
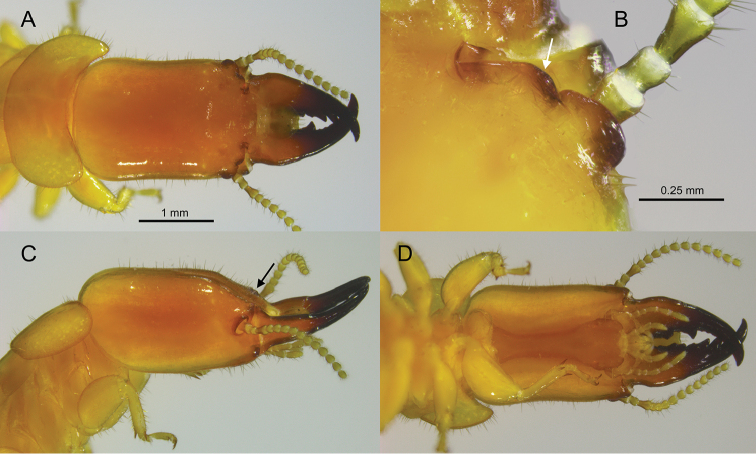
Head and pronotum of the *Rugitermes aridus* soldier **A** dorsal **B** right (arrow pointing to outer margin of anterolateral ridge) **C** lateral (arrow pointing to antennal carinae) **D** ventral.

**Figure 3.**
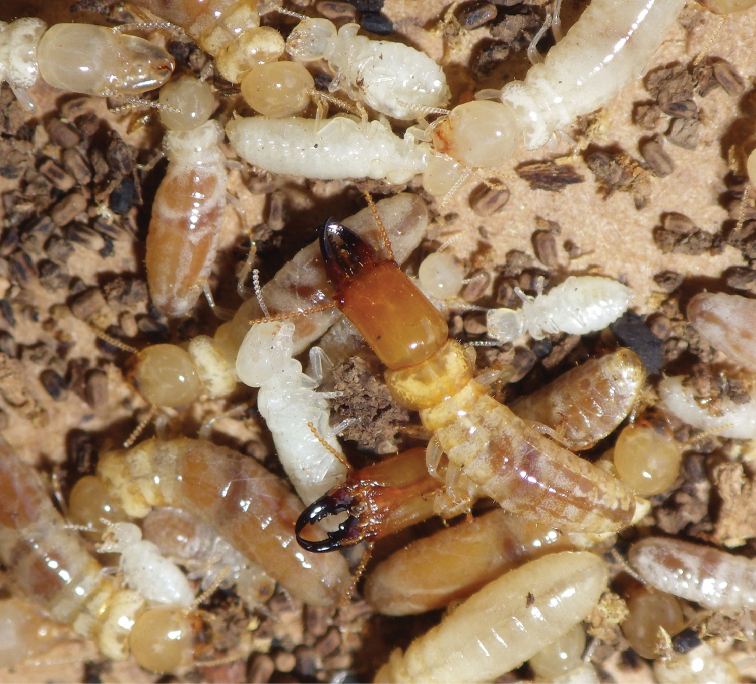
Field habitus of a soldier and pseudergates of *Rugitermes rufus*.

Figures 2 , 3 ; Table 1

#### Diagnosis.

The *R. aridus* soldier is much smaller than that of *R. laticollis*.

The antennal carinae and anterolateral ridges of the *R. aridus* soldier are smoothly rounded, modestly sclerotized, and appear polished; while these structures in *R. rufus* and *R. volcanensis* are more angular, darkly sclerotized, and rugose. In *R. aridus*, the soldier head capsule width at the antenna is narrower than span of antennal carinae while in in *R. rufus* and *R. volcanensis* this span is equal to the width of the head capsule. Also, in *R. aridus*, the outside corners of the anterolateral ridges are not elevated above the plane of the frons as they are in *R. rufus* and *R. volcanensis*.

#### Type locality.

Peru, Huánuco, 10 km NE Huánuco city (Fig. 9A).

#### Material examined.

***Holotype*** soldier: Peru, Huánuco, 10 km NE Huánuco city (-9.877, -76.1641), elev. 2127 m, 1 Jun. 2014, R. Scheffrahn et al. (R. Scheffrahn, T. Carrijo, J. Chase, J. Křeček, E. Kuswanto, J. Mangold, A. Mullins, and T. Nishimura), University of Florida Termite Collection (UFTC), Davie Florida, no. PU991. ***Paratypes*.** One additional soldier, pseudergates, same colony sample as holotype. Two additional colonies from type locality (same data), one soldier and pseudergates (PU990) and two soldiers and pseudergates (PU992). Five colonies: Peru, Huánuco, 7 km NE Huánuco city (-9.8816, -76.1913), elev. 1926 m, 1 Jun. 2014, R. Scheffrahn et al., University of Florida Termite Collection nos. PU997, PU998, PU999, PU1000, and PU1001, each containing two soldiers and pseudergates.

**Table 1.** Measurements (mm) of *Rugitermes* soldiers.

Character	*R. aridus*	*R. aridus*	*R. rufus*	*R. rufus*	*R. volcanensis*	*R. volcanensis*
	Range	Mean	Range	Mean	Range	Mean
Head length to lateral mandible base	2.13–2.69	2.39	1.72–2.72	2.33	2.32–2.88	2.62
Head width, maximum	1.44–1.72	1.6	1.16–1.56	1.42	1.24–1.88	1.54
Head height with gula, max.	1.15–1.45	1.33	0.90–1.32	1.18	1.12–1.38	1.26
Pronotum length	0.98–1.25	1.13	0.64–1.05	0.91	1.00–1.25	1.08
Pronotum width	1.60–2.03	1.76	1.10–2.10	1.56	1.50–1.90	1.63
No. antennal articles	11–16	13.7	12–13	12.43	10–16	14
3^rd^ antennal article length	0.18–0.25	0.21	0.14–0.22	0.18	0.16–0.25	0.19
*n*	15		14		8	
No. colonies	8		4		4	

#### Description.

***Imago*** (unknown).

***Soldier*** (Fig. 2). Head capsule, in dorsal view, light orange-brown from frons to mid-vertex, then grading to light yellow-brown at occiput; anterior of mandibles nearly black, becoming orange-brown mid-length to base. Antennal carinae, anterolateral ridges, and genal horn dark brown. In ventral view, postmentum orange-brown, head capsule light yellow-brown. Head capsule long, rectangular; lateral margins noticeably concave in middle, covered evenly with setae except at occiput. Pronotum much wider than long; with scattered setae, denser along lateral margins; anterior margin weakly concave. Frontal flange forming shallow furrow; angled 30° with plane of vertex. In lateral view (Fig. 2C), outside corners of the anterolateral ridges are not elevated above the plane of the frons. Antennal carinae and anterolateral ridges glabrous, smoothly rounded. The outer margins of each ridge curve to form angles of ca 120°. Eye spots small, narrowly oblique, barely lighter than head capsule. Third antennal article club-shaped, about twice as long as second and fourth articles. Mandibles about two-thirds length of head capsule; tip curves about 60°; outer margin of mid-blade straight, large shallow hump at base. Measurements of 15 soldiers from eight colonies are shown in Table 1.

#### Distribution

(Fig. 1). Upper Huallaga river valley in vicinity of Huánuco, Peru.

#### Etymology.

The species name “aridus” describes the arid habitat where this species lives.

### Rugitermes rufus

Taxon classificationAnimaliaIsopteraKalotermitidae

Scheffrahn sp. nov.

E821B832-2637-5A5C-BC17-713977CA1C57

http://zoobank.org/F4999D2D-8839-43CE-9C91-BDBDC146C59B

**Figure 4.**
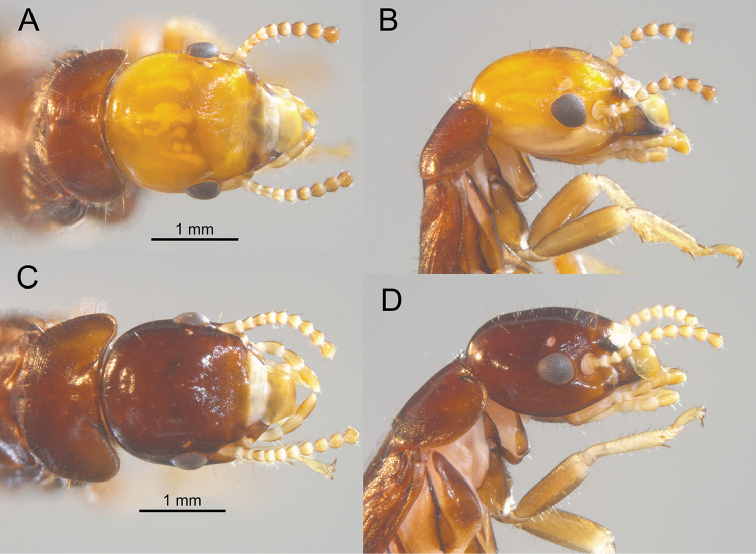
Head and thorax of the *Rugitermes rufus* (**A** dorsal **B** lateral) and the *R. volcanensis* (**C** dorsal **D** lateral) imagos.

**Figure 5.**
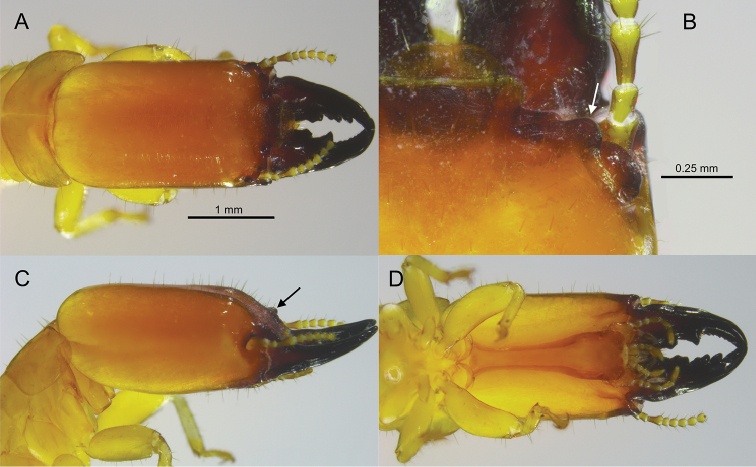
Head and pronotum of the *Rugitermes rufus* soldier **A** dorsal **B** right (arrow pointing to outer margin of anterolateral ridge) **C** lateral (arrow pointing to antennal carinae) **D** ventral.

**Figure 6.**
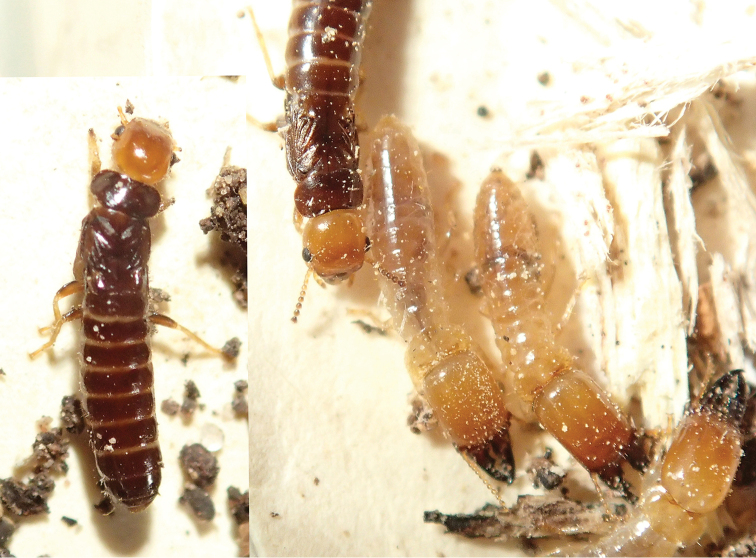
Field habitus of the imago and soldiers of *Rugitermes rufus*.

Figures 4A, B , 5 , 6 ; Tables 1 , 2

#### Diagnosis.

**Figure 7.**
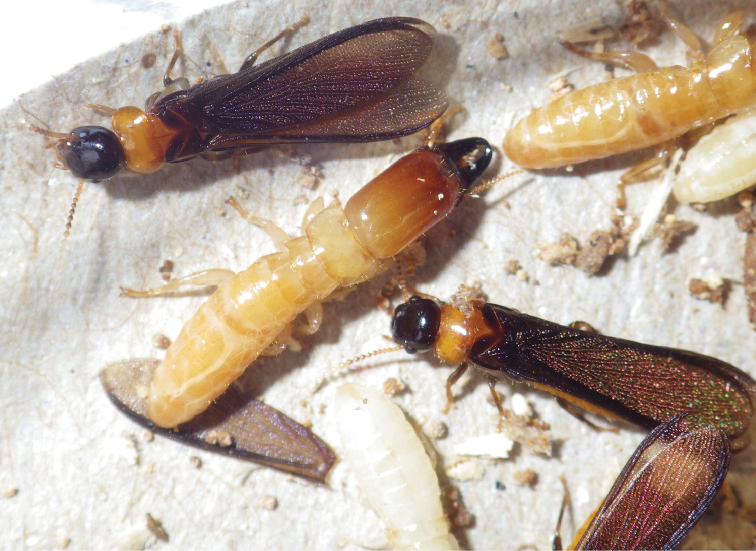
Field habitus of the imagos and soldiers of *Rugitermes magninotus* from Peru (UFTC no. PU222).

The coloration and size of the *R. rufus* imago easily distinguish it from all other species of *Rugitermes*. *Rugitermes rufus* is the only *Rugitermes* imago with a reddish-orange head capsule and dark brown coloration of the pronotum and remainder of the body. *Rugitermes costaricensis* (Snyder, 1929) and *R. unicolor* Snyder, 1952 imagos each have a yellow-brown head and yellow pronotum (with less contrast between coloration of head and pronotum in *R. unicolor*). In all other *Rugitermes*, the imago head is black and the pronotum is yellow [*R. bicolor* (Emerson, 1926), *R. flavicinctus* (Emerson, 1925), *R. magninotus* (Fig. 7), *R. kirbyi* (Snyder, 1926), *R. nodulosus* Oliveira, 1979, *R. panamae* (Snyder, 1925), and *R. rugosus* (Hagen, 1858)] or the head and pronotum are both black [(*R. athertoni*, *R. laticollis*, *R. niger* Oliveira, 1979, and *R. occidentalis* (Silvestri, 1901)]. The head width of the *R. rufus* imago is among the smallest: in range of *R. flavicinctus* (1.2 mm) and *R. magninotus* (1.25 mm). The soldier of *R. rufus* is smaller in all measurements compared with *R. volcanensis*. The antennal carina in *R. rufus* forms a smaller ovoid shelf of the gena and, in lateral oblique view, projects acutely from the frontal margin. In *R. volcanensis*, the shelf formed by the antennal carina is larger and, in the lateral oblique view, weakly projects above the frontal margin. The third antennal article of *R. rufus* is proportionally narrower and longer than that of *R. volcanensis*. See also diagnosis of *R. aridus*.

#### Type locality.

Bolivia, Amboró National Park, Refugio Los Volcanes, Santa Cruz District (Fig. 9B).

#### Material examined.

***Holotype.*** One female delate imago: Bolivia, Amboró National Park, hillside above Refugio Los Volcanes, Santa Cruz District (-18.119680, -63.608810) elev. 1402 m, 2 Jun. 2014, R. Scheffrahn et al., UFTC no. B01021**. *Paratypes*.** Three soldiers, pseudergates, same colony sample as holotype. Five soldiers, pseudergates, Bolivia, Amboró National Park, forest surrounding Refugio Los Volcanes, Santa Cruz District (-18.1004, -63.5934) elev. 1374 m, 3 Jun. 2014, R. Scheffrahn et al., UFTC no. BO1055. Four colonies, same locality as BO1055: three soldiers, pseudergates (BO1061), four soldiers, pseudergates (BO1062), two male dealate imagos, pseudergates (BO1070).

#### Description.

***Dealated imago*** (Figs 4A, B, 6). Head capsule reddish orange; pronotum, thorax, abdomen, and wing scales concolorous dark brown. Compound eye small; trapezoidal; with shorter straight margin below ocellus and more curved margin near gena. Ocellus ovoid, concolorous with interior of antennal socket; one-half diameter removed from eye margin. Head vertex and frons covered with about a dozen erect setae ca 0.15-mm-long; frons darker, with faint rugosity at frontal flange. Pronotum slightly wider than head capsule; anterior margin slightly incised; anterolateral corners nearly rectate, posterior margin narrowly concave; margins with few longer and many shorter setae. Pronotum interior pilosity congruent with vertex. Antennae with basal article relative lengths 1>2=3<4. Wing scale covered with about 20 setae of similar length and density as those on head and pronotum. Legs with femora light orange-brown; tibia much lighter. Arolium present. Measurements of three imagoes from two colonies are shown in Table 2.

**Table 2.** Measurements (mm) of *Rugitermes* dealated imagos.

Character	*R. rufus*	*R. rufus*	*R. volcanensis*
	Range	Mean	
Head width, maximum (w/eyes)	1.19–1.26	1.23	1.21
Pronotum width, max.	1.21–1.29	1.24	1.27
Eye diam. ocellus, max.	0.08–0.10	0.09	0.07
Eye diam. compound, max.	0.27–0.30	0.28	0.27
Body length	6.00–6.90	6.33	7.2
Right forewing length			
Body length with wings			
No. antennal articles, max.	9 (broken)		10 (broken)
*n*	3		1
No. colonies	2		1

***Soldier*** (Figs 5, 6). Head capsule, in dorsal view, dark brown along margin of anteclypeus, anterolateral ridges, and antennal carinae grading to orange brown at frons, orange at mid-capsule and yellow at occiput; mandibles nearly black throughout. In ventral view, postmentum light orange-brown contrasting with light yellowish head capsule. Head capsule long, rectangular; lateral margins parallel, covered with a few setae except at occiput. Pronotum much wider than long; with a few long setae at margins and middle. Frontal flange and frons forming shallow furrow; angled 40° with plane of vertex. In dorsal view, antennal carina forms small ovoid shelf over the gena. In lateral oblique view, antennal carinae project acutely from the lateral margin of frons (Fig. 5C). Antennal carinae and anterolateral ridges weakly rugose. The outer margin of each ridge form angle of ca 110° (Fig. 5B). Eye spots large, narrowly oblique, barely lighter than head capsule. Third antennal article narrow at the base and longer than second and fourth article combined. Mandibles about one-half length of head capsule; tip curves about 60°; outer margin of mid-blade slightly curved, shallow hump at base. Measurements of 14 soldiers from four colonies are shown in Table 1.

#### Distribution.

(Fig. 1). Amboró National Park, a transition of the Tucuman-Bolivian forest and the montane Chaco.

#### Etymology.

The species name “rufus” describes the unique reddish-orange coloration of the imago head.

### Rugitermes volcanensis

Taxon classificationAnimaliaIsopteraKalotermitidae

Scheffrahn sp. nov.

7328C3D1-942A-532C-8556-CE0C6EF54FF8

http://zoobank.org/6A431AE6-45D2-4911-8631-8802F0DF6A28

**Figure 8.**
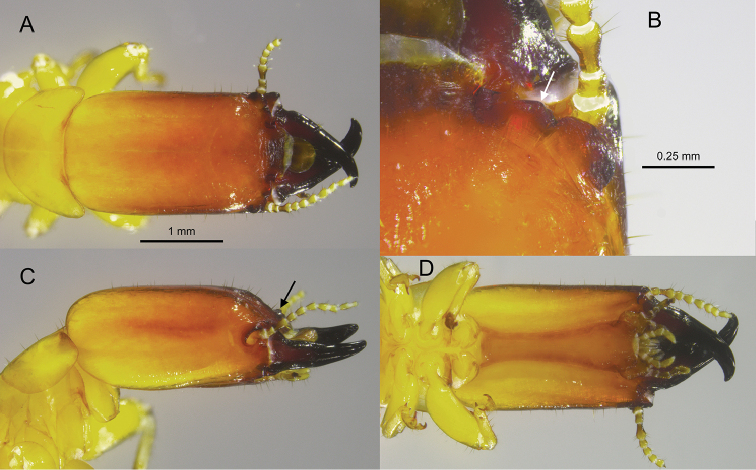
Head and pronotum of the *Rugitermes volcanensis* soldier **A** dorsal **B** right (arrow pointing to outer margin of anterolateral ridge) **C** lateral (arrow pointing to antennal carinae) **D** ventral.

Figures 4C, D , 8 ; Tables 1 , 2

#### Diagnosis.

See the diagnoses of *R. aridus* and *R. rufus* above.

#### Type locality.

**Figure 9.**
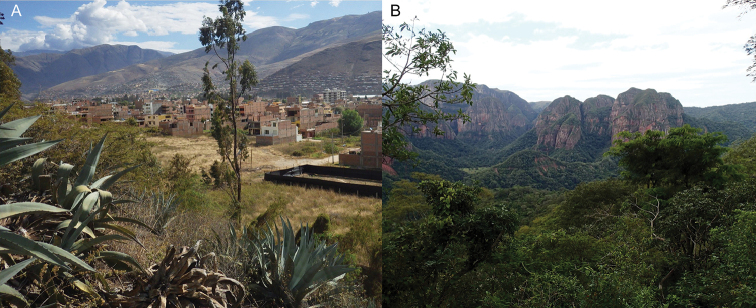
Biomes for new *Rugitermes***A***R. aridus***B***R. rufus* and *R. volcanensis*.

Bolivia, Amboró National Park, Refugio Los Volcanes, Santa Cruz District (Fig. 9B).

#### Material examined.

***Holotype.*** One male delate imago (with pseudergates): Bolivia, Amboró National Park, forest surrounding Refugio Los Volcanes, Santa Cruz District (-18.119680, -63.608810) elev. 1402 m, 2 Jun. 2014, R. Scheffrahn et al., UFTC no. B01069**. *Paratypes.*** Bolivia, Amboró National Park, hillside above Refugio Los Volcanes, Santa Cruz District (-18.119680, -63.608810) elev. 1402 m, 2 Jun. 2014, R. Scheffrahn et al., B01022. Two colonies, same locality as BO1069: five soldiers, pseudergates (BO1117), one soldier, pseudergates (BO1118).

#### Description.

***Dealated imago*** (Fig. 4C, D). Head capsule, pronotum, thorax, abdomen, and wing scales concolorous chestnut brown. Compound eye small, elliptical. Ocellus very small, ovoid, hyaline coloration contrasting with brown head capsule; three-quarters diameter removed from eye margin. Head vertex and frons covered with over a dozen erect setae ca 0.10–0.20-mm-long; with faint rugosity at frontal flange. Pronotum wider than head capsule; anterior margin weakly concave; anterolateral corners nearly rectate, posterior margin slightly incised in middle; margins with few longer and many shorter setae. Pronotum interior with a few long setae. Antennae with basal article relative lengths 1>2<3>4. Wing scale covered with few short setae. Legs with femora light brown; tibia much lighter. Arolium present. Measurements of the holotype imago are shown in Table 2.

***Soldier*** (Fig. 8). Head capsule, in dorsal view, brown along margin of anteclypeus, anterolateral ridges, and antennal carinae grading to orange from frons to mid-capsule and yellow in posterior half; mandibles nearly black throughout. In ventral view, postmentum light orange-brown contrasting with light yellowish head capsule. Head capsule long, rectangular; lateral margins with very slight convergence, covered with a few setae except at occiput. Pronotum wider than long with a few long setae at margins and middle. Frontal flange and frons forming shallow furrow; angled 40° with plane of vertex. In dorsal view, antennal carina forms ovoid shelf eclipsing over half of the first antennal article. In lateral oblique view, antennal carinae project obtusely from the lower lateral margin of frons (Fig. 7C). Antennal carinae and anterolateral ridges moderately rugose. The outer margin of each ridge forms angle of ca 140° (Fig. 7B). Eye spots large, narrowly oblique, barely lighter than head capsule. Third antennal article nearly as long as second and fourth article combined. Mandibles slightly less than one-half length of head capsule; tip curves about 60°; outer margin of mid-blade straight, very shallow hump at base. Measurements of eight soldiers from four colonies are shown in Table 1.

#### Distribution.

(Fig. 1). Same as *R. rufus*.

#### Etymology.

The species name “volcanensis” refers to the scenic type locality of Refugio Los Volcanes.

#### Phylogenetic and DNA barcode analysis.

**Figure 10.**
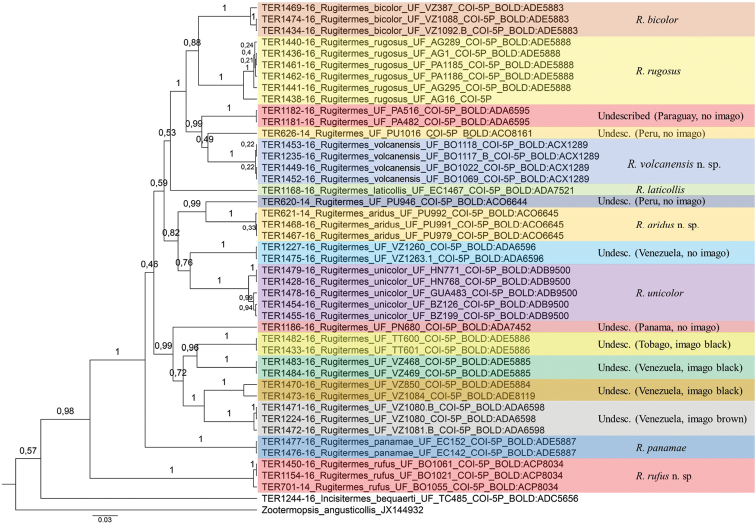
Bayesian phylogenetic tree of Neotropical *Rugitermes* using the COI region. Branch support is posterior probability. Different colors show the 17 recognized species by the GMYC analysis.

Our phylogenetic analysis recovered the new species *R. rufus* as sister group of all other *Rugitermes*. The new species *R. volcanensis* was clustered with *R. bicolor*, *R. rugosus*, and two undescribed species from Peru and Paraguay. *Rugitermes aridus* sp. nov. is more related to an undescribed species from Peru. Our analysis also recovered a clade of five undescribed species from northern South America and Central America (Fig. 10).

In total, 17 entities were recognized as species according to the GMYC barcode analysis (different colors in Fig. 10; confidence interval: 10–23; Likelihood ratio test > 0.0001). *Rugitermes bicolor*, *R. laticollis*, *R. panamae*, *R. rugosus*, and *R. unicolor* were corroborated by the analysis, as well as the three new species described here and other nine still undescribed species.

## Discussion

Most *Rugitermes* soldiers are difficult to distinguish from each other, but the anterolateral ridges of the frons are helpful (Scheffrahn and Florian 2020). Therefore, the imago caste is often valuable for correct identification.

*Rugitermes aridus* occurs in the rain-shadowed upper Huallaga river valley of Peru (Fig. 9A). Here the temperature is cool and stable year-round (22°C daytime, 13°C at night) characterized by cacti and other xeric flora. The only other termite species we encountered in this area was *Cryptotermes brevis* (Walker, 1853). As with *R. aridus*, specimens of *C. brevis* were collected from *Acacia* and *Prosopis* trunks and branches. This marks a >200 km inland endemic range extension for *C. brevis* from that reported by Scheffrahn et al. (2009). Conversely, the area of southeastern Amboró National Park, Bolivia, where *R. rufus* and *R. volcanensis* were collected, constitutes a mesic forest (Fig. 9B). It has termite elements from Chaco [e.g., *Tauritermes taurocephalus* (Silvestri, 1901)], Atlantic Forest [e.g., *Procornitermes lespesii* (Müller, 1873)], Andean montane [(e.g., *Comatermes perfectus* (Hagen, 1858), *Dolichorhinotermes lanciarius* Engel & Krishna, 2007)], and Amazonia/Cerrado [(e.g., *Diversitermes diversimilis* (Silvestri, 1901), *Nasutitermes ephratae* (Holmgren, 1910)].

The DNA barcode analysis shows a high undescribed diversity of *Rugitermes* in the New World. As well as many other kalotermitids, this group still need extensive taxonomic work. Different from most other termite genera, in which soldiers are the most important caste for taxonomic purposes, *Rugitermes* identification and diagnosis are much more reliable using the imago caste. Since this caste is absent in many samples, this is yet another challenge for the taxonomy of the group.

## Supplementary Material

XML Treatment for Rugitermes aridus

XML Treatment for Rugitermes rufus

XML Treatment for Rugitermes volcanensis

## References

[B1] Darriba D Taboada GL Doallo R Posada D (2012) jModelTest 2: more models, new heuristics and parallel computing. Nature Methods 9: 772. 10.1038/nmeth.2109 PMC4594756 22847109

[B2] deWaard JR Ivanova NV Hajibabaei M Hebert PDN (2008) Assembling DNA barcodes: analytical protocols. In: Martin C (Ed.) Methods in Molecular Biology: Environmental Genetics.Humana Press, Totowa, 275–293. 10.1007/978-1-59745-548-0_15 18642605

[B3] Drummond AJ Suchard MA Xie D Rambaut A (2012) Bayesian phylogenetics with BEAUti and the BEAST 1.7. Molecular Biology and Evolution 29: 1969–1973. 10.1093/molbev/mss075 22367748 PMC3408070

[B4] Fujisawa T Barraclough TG (2013) Delimiting species using single-locus data and the generalized mixed Yule coalescent approach: a revised method and evaluation on simulated data sets. Systematic Biology 62: 707–724. 10.1093/sysbio/syt033 23681854 PMC3739884

[B5] Hebert PDN Cywinska A Ball S deWaard J (2003) Biological identifications through DNA barcodes. Proceedings of the Royal Society of London series B – Biological Sciences 270: 313–321. 10.1098/rspb.2002.2218 12614582 PMC1691236

[B6] Krishna K Grimaldi DA Krishna V Engel MS (2013) Treatise on the Isoptera of the world: Volume 2 Basal Families. Bulletin of the American Museum of Natural History 377: 203–623. 10.1206/377.2

[B7] Light SF (1932) Termites of the Marquesas Islands. Bulletin of the Bernice P. Bishop Museum 98: 73–86. [+ 3 pls]

[B8] R Core Team (2018) R: a language and environment for statistical computing. R Foundation for Statistical Computing, Vienna. https://www.R-project.org/

[B9] Rambaut A Suchard MA Xie D Drummond AJ (2014) Tracer v1.6. http://beast.community/tracer

[B10] Scheffrahn RH (2015) Global elevational, latitudinal, and climatic limits for termites and the redescription of *Rugitermes laticollis* Snyder (Isoptera: Kalotermitidae) from the Andean Highlands. Sociobiology 62: 426–438. 10.13102/sociobiology.v62i3.793

[B11] Scheffrahn RH (2019a) Expanded New World distributions of genera in the termite family Kalotermitidae. Sociobiology 66: 136–153. 10.13102/sociobiology.v66i1.3492

[B12] Scheffrahn RH (2019b) UF Termite database. University of Florida termite collection. https://www.termitediversity.org/ [Accessed on: 2020-25-10]

[B13] Scheffrahn RH Florian OPP (2020) *Rugitermes tinto*: a new termite (Isoptera, Kalotermitidae) from the Andean region of Colombia. ZooKeys 963: 37–44. 10.3897/zookeys.963.55843 32922130 PMC7458932

[B14] Scheffrahn RH Křeček J Ripa R Luppichini P (2009) Endemic origin and vast anthropogenic dispersal of the West Indian drywood termite. Biological Invasions 11: 787–799. 10.1007/s10530-008-9293-3

[B15] Yule GU (1925) II. A mathematical theory of evolution, based on the conclusions of Dr. JC Willis, FR S. Philosophical Transactions of the Royal Society of London – Series B, containing papers of a biological character 213: 21–87. 10.1098/rstb.1925.0002

